# Surgery

**Published:** 1861-03

**Authors:** S. W. Gross

**Affiliations:** Lecturer on Surgical Anatomy and Operative Surgery, Philadelphia


### Bi-Monthly Abstracts;

OR, BI-MONTHLY NOVELTIES IN MEDICAL, PATHOLOGICAL, SURGICAL, OBSTETRICAL, PHYSIOLOGICAL, AND CHEMICAL SCIENCE.

### SURGERY.

BY S. W. GROSS, M.D.,
LECTURER ON SURGICAL ANATOMY AND OPERATIVE SURGERY, PHILADELPHIA.

I.—*On Lead Amaurosis.* By ROBERT SCOTT ORR, M.D. (Glasgow Medical Journal, vol. viii. No. 31, 1860.)

DR. ORR having met with an instance of amaurosis, the result of lead poisoning, makes it the basis of a very interesting paper, and has collected twenty-seven cases, all that he was able to find recorded, of this rare and singular affection. The subject was very fully discussed, nearly a quarter of a century ago, by Tanquerel, and from his treatise on the diseases resulting from the toxic properties of lead the author has collected many of the facts contained in his communication.

The patient of Dr. Orr was a boy, twelve years of age, of a scrofulous habit, who was admitted into the Glasgow Royal Infirmary on the 29th of June, 1860, laboring under the symptoms of lead poisoning. For three years he had been working in a pottery, and for two-thirds of this period he worked in clay, during which time his health was excellent. At the commencement of the third year he was transferred to the glazing department, and in a few months his general health began to suffer. After the lapse of seven months, he had an attack of lead colic, and also another attack six months previous to his admission to the infirmary, and for the last four months his friends noticed a well-marked blue line along the margin of the gums. On the seventh of June he felt cold and uncomfortable, and cerebral symptoms manifested themselves, consisting in four very severe epileptic convulsions, each of about a minute's duration, and all occurring within about ten hours, and succeeded by a state of unconsciousness, which lasted about fifty hours. For several days after this, his mind wandered, he complained of pain in the abdomen, but was able to walk about. On the fourteenth the first indications of paralysis were noticed, and these gradually increased; intense headache then supervened. On admission, the patient was much debilitated, his limbs were partially paralyzed, and the muscles, especially those of the upper extremities, were much wasted. For two days he had been almost totally blind; the eyes were very prominent and the pupils widely dilated. He still complained of pain in his head, but he was quite sensible, and his memory was unimpared. The left facial nerve was paralyzed, the corresponding side of the face being fixed and devoid of expression. In about two weeks the third nerve became affected, as was shown by the existence of ptosis, the conjunctiva was inflamed, and was the seat of a copious purulent discharge. After the lapse of six weeks he was much stronger and stouter, the facial paralysis and ptosis had nearly disappeared, but the eyes remained fixed and motionless, and his vision was not at all improved.

An examination with the ophthalmoscope revealed apoplexy of the retina, its vessels being much congested, and its surface covered with ecchymosed patches. The treatment in this case consisted, at first, in sustaining the patient's strength with wine and nourishing diet, and in the application of a small blister to the nape of the neck, together with the internal administration of the iodide of potassium, which was, however, discontinued for a time, but was afterward resumed, and continued throughout the remainder of the treatment. Active purgation was not at first employed, for fear of increasing the debility; but the bowels were attended to, and, as soon as his strength permitted, cathartics were freely administered. His stools were black, and he always felt much better after each motion. Opium had a happy effect in procuring rest and sleep, and nux vomica was given for the paralysis of the face and limbs. The facial palsy seemed to have been benefited by free counter-irritation to neck and behind the ears, and a seton was also inserted in the nape of the neck. On the fourteenth of September the patient continued amaurotic, but his general health was much improved. The probability is that he will remain permanently blind.

In reviewing the twenty-seven recorded cases of lead amaurosis, Dr. Orr divides them into three classes: First, those in which the amaurosis preceded other forms of lead affection, and was the first indication of the effect of lead in the system; second, those in which the amaurosis was preceded by colic, constipation, articular pains, and paralysis only; and third, those in which it was ushered in not only by colic, articular pains, and paralysis, but also by various head symptoms, such as delirium, epilepsy, convulsions, and coma, constituting what Tanquerel has designated encephalopathy.

In the first class of cases, or those in which amaurosis was the first indication of lead in the system, we find but five cases recorded, and four of these are remarkable from the fact that the patients had been exposed to the effects of lead, for periods varying from six to twenty years, before they became amaurotic. Under the second class thirteen cases are enumerated, and under the third nine cases, in which is included the case of the author. The duration of lead amaurosis is recorded in sixteen cases, which ultimately recovered. In seven it lasted from one to nine days; in four, for about a fortnight; and in five, from nineteen to seventy days.

II.—*Wound of the Spinal Cord, followed by Recovery.* By M. PRESTAT. (Gazette Hebdomadaire, No. 49, 1860.)

A boy, fifteen years of age, was wounded with a knife in the lower dorsal region, and immediately fell down, remaining for some time in the recumbent posture, from inability to rise. When seen, four hours afterward, M. Prestat found that the knife had penetrated the cord between the laminæ of the tenth and eleventh dorsal vertebræ. There had been little hemorrhage, but a large quantity of a limpid, serous fluid had escaped from the wound. The expression of the youth was denotive of great suffering, the pulse was small, the skin cold, the intellect clear, and respiration was carried on in the most perfect manner. He had vomited once, but there was no involuntary escape of urine, nor of fecal matter, and the lower extremities were paralyzed—the left completely, the right partially. During the evening of the same day, the warmth of surface had returned, and the pulse became natural. He was bled at this time, as well as on the following day, and for several days it was found necessary to evacuate the bladder with a catheter, a purgative enema being administered at the end of a week. During this period the wound remained open, and gave issue to a large quantity of cerebro-spinal fluid.

The patient soon regained control over his bladder, and the wound began to cicatrize. Somewhat later, defecation became easy, but the paralysis persisted, and the left leg became atrophied. About a month after the injury M. Prestat had recourse to electricity, under the use of which motion and sensation gradually returned. By degrees the cure was complete, without any trace of the lesion remaining.

III.—*Undescribed Malformation of the Lower Lip Occurring in Four Members of One Family.* By J. JARDINE MURRAY, F.R.C.S.E. (British and Foreign Medico-Chirurgical Review, October, 1860.)

The congenital malformation described by Mr. Murray consists in the presence of two sacculi or pouches in the lower lip of the father, the first, third, and eighth children of the same family, their ages being respectively forty-one, seventeen, thirteen, and one and a half years. The pouches in all these cases are exactly similar, and the description of one will suffice for the rest. The under lip is prominent and fleshy, and about a quarter of an inch from the external edge of the mucous membrane of the prolabium are two crescentic openings, one on each side of the median line, the horns of the crescent being directed forward and a little outward. A probe passes to the depth of half an inch under the thick mucous membrane, and each sacculus can readily admit a split pea. They secrete a glairy mucus, do not communicate with each other, nor does their presence produce any inconvenience.

Mr. Murray, as well as many other medical men, is unable to account for the existence of this curious affection, but inclines to the opinion that it may be explained by some peculiarity in the development of the frænum, or that it is the result of intra-uterine disease of the labial glands. Besides the pouches of the lower lip, other defects of formation exist among the members of the same family, to a striking extent, and show in a remarkable manner the hereditary nature of malformations. The mother, and her second, fourth, and sixth children are perfectly well formed. The father's mother, aged sixty-five, has a very narrow and much arched palate. The father, aged forty-one, has two sacculi in the lower lip, and was operated on for a double hare-lip by the late Mr. Liston. The first child, a girl, aged seventeen, has two pouches in the lower lip. The third child, a girl, aged thirteen, has a sacculated under lip, and was operated on for double hare-lip in infancy. The fifth child, aged nine, has the palate much arched and very narrow. The seventh child, aged three, has the ring and middle fingers of both hands webbed; and the eighth child, aged eighteen months, has a sacculated lower lip, and was relieved of a single hare-lip.

IV.—*Sebaceous Tumors Simulating External Piles.* By Professor Hyrtl. (Journal de Médecine de Bordeaux, No. 11, 1860.)

Professor Hyrtl having been affected for eight days with two round tumors, about the size of a pea, situated near the margin of the anus, believed himself to be the subject of external piles. They were free from pain, did not give rise to the slightest inconvenience, nor were they influenced by cold ablutions. Having a great desire to see them, he made use of two mirrors, and to his astonishment found that the tumors were white, instead of being blue as in the case of hemorrhoids. This fact led him to fear the existence of some malignant disease, and on raising them one day, as he often did, to assure himself that they were not adherent to the deeper structures, he detached a soft, round body, which was found, under the microscope, to consist of corrugated sebaceous cells, a large quantity of fat, granular matter, and epithelial scales, with some cholesterine. He was now aware that he had to deal with sebaceous tumors, and inasmuch as the sebaceous follicles situated about the anus are devoid of muscular fibres, they evinced a great tendency to refill, which was, however, prevented by compression. Dr. Hyrtl does not enter fully into the anatomy of these follicles, simply stating that the largest are situated nearest the anus and that several open upon the mucous membrane of the rectum. Simple compression will suffice, in every case, for the cure of these tumors.

V.—*Ovariotomy in London Hospitals.* (British Medical Journal, Dec. 1st, and Dec. 8th, 1860.)

Between January, 1858, and November, 1860, twenty-eight operations for ovarian tumors have been performed in London hospitals, the general result being fifteen deaths, against thirteen recoveries. In two of the recoveries from the operations, the patients died some months afterward of cancer, and eleven of the recoveries have been permanent, the subjects being now strong and well. Twelve of these cases occurred to Mr. Spencer Wells, in the Samaritan Hospital, the result of his experience in hospital practice being eight recoveries against four deaths.

Between February, 1858, and October, 1860, (*Medical Times and Gazette*, December 1st, 1860,) Mr. Wells has operated in twenty cases, twelve, as stated above, being in the Samaritan Hospital, the remaining eight occurring in his private practice. Of the latter, five were successful and three terminated fatally. These statistics, although small, undoubtedly prove that ovariotomy is justifiable in certain cases.

VI.—*On the Preventive Treatment of Ectropion by Temporary Closure of the Lids.* By M. Debrou. (Gazette Hebdomadaire, No. 45, 1860.)

In a communication to the Surgical Society of Paris, M. Debrou calls attention to the prevention of ectropion, consequent upon malignant pustule, by closure of the eyelids. So soon as the slough has been cast off, a large granulating wound is left, and during the process of cicatrization, the parts contract to so great a degree as to evert the lid, thus necessitating a plastic operation for its restoration.

In order to prevent this deformity, he places the lids in such a condition as that the contracting force cannot act upon the one likely to become involved. This he accomplishes by the production of an ankyloblepharon, which is unattended with bad consequences, since, when it is destroyed, the sight is found to be perfectly good, notwithstanding the eye has been deprived of rays of light for a longer or shorter period. The operation is very simple, consisting merely in removing with the scissors a small portion of the free border of each lid, being careful to avoid the lashes. The raw surfaces are then brought together with from four to six points of suture, and union is almost always obtained. In order to permit of the escape of tears and mucus, as well as to admit of the introduction of a delicate grooved director, upon which the interciliary cicatrix can readily be divided without danger to the globe of the eye, when the cure is effected, M. Debrou advises that both angles of the eye should be left a little open.

In regard to the length of time the lids should be kept closed, it is impossible to give a definite answer. They should rarely be separated before the third month; sometimes not even for one year; in general terms the union is to be maintained until the loss of palpebral substance is completely repaired and has undergone as much contraction as is possible.

VII.—*Spontaneous Cure of Cataract.* By ST. GEORGE PEACHY, M.D. (Maryland and Virginia Medical Journal, February, 1861.)

Mr. M., fifty years of age, of correct habits, and accustomed to exercise a great deal in the open air, was the subject, in 1835, of inflammation of the left eye, attended with the most intense pain. The inflammation was subdued in the course of time, but he suffered occasional pain, and the sight of the right eye became impaired. In a few years the left eye became completely cataractous, but he was still able to see quite well with the right, so that an operation was not deemed advisable. The left globe was also unusually prominent. Dr. Peachy thus concludes the imperfect account of the case. "Several months ago, Mr. M. felt a renewal of the fullness, he first spoke of in the globe of the eye—pain accompanied it, and suddenly he felt 'something give way.' To his astonishment, upon raising the lid, his sight had been restored to him as if by miracle, and for six months he has enjoyed as perfect vision as ever. In proof of this last assertion, in the presence of myself and others, he has read with the left eye alone, an ordinary newspaper print with perfect ease and without the aid of glasses. The lens escaped into the anterior chamber, fell below the axis of vision, and has been undergoing gradual absorption. It was a hard, lenticular cataract, judging from its size and the amber color so frequently mentioned by writers on this disease. The right eye has recovered its normal vigor, and Mr. M. has now perfect vision and no pain. Excepting the small amber-colored body resting against the lower margin of the cornea, within the anterior chamber, no one would discover that Mr. M. had suffered from any such affection as that of which I have given you the above description, and of which I can find no such termination in any work in my possession."

VIII.—*On Lithotomy in Persia.* By Dr. POLLAK, of Teheran, Persia. (Edinburgh Medical Journal, January, 1861.)

The Vienna correspondent of the *Edinburgh Medical Journal* gives an account of a very interesting communication made to the Royal Society of Physicians, by Dr. Pollak, the private physician of the Shah of Persia, "on 158 lithotomy operations performed in Persia between November, 1852, and June, 1860." Lithiasis is very common in Persia, and is usually confounded with gonorrhœa. Men are more frequently affected than women, the rich and poor are equally subject to it, and the malady is rarely seen among the Jews and Armenians, and the author has never witnessed it in the negro race. In regard to the etiology of vesical calculus, Dr. Pollak thinks it mainly due to the immoderate use of milk diet, especially sour milk, and to the use of sour, unripe fruit. Another cause may be the inordinate eating of rice, and it is less likely to occur in those who use a flesh diet and drink alcoholic wines. The fact that renal and vesical calculi are frequently found in the mountain sheep of Persia seems to favor the opinion of a purely vegetable diet being concerned in producing the disease. The greatest number of cases occur during early life: thus of 158 patients cut by Dr. Pollak, 69 were between the ages of 1 and 7 years; 53 between 8 and 14; 9 between 15 and 21; 16 between 21 and 50; and 11 above 50. The frequent occurrence of calculous disease in childhood is ascribed by the author to prolonged suckling, the usual period of lactation being two years, and often for a longer time.

The lateral operation was performed in 121 cases, of which only seven died. In two of the cases the wounds healed by the first intention, and the patients were discharged at the end of the sixth day. When the wounds united by granulation, the process was usually complete by the fourteenth or fifteenth day. Surgical fever was rarely seen, and there was never any occasion to restrict the diet after the third day. Only twice did he operate by the rectum, and then for small stones impacted in the prostate. Dr. Pollak always operated in the open air, never in a room, and almost invariably employed chloroform as an anæsthetic. In 147 cases he found but one calculus in the bladder; in one case there were six, in two cases four, and in eight cases two stones. Dr. Pollak ascribes the very favorable results of his operations to the following circumstances: 1st. The sound constitutions of the patients. 2d. The operations being invariably performed in the open air, and the after-treatment being conducted either out of doors or in a chamber with open doors and windows. 3d. The absence of erysipelas. 4th. Dressing the wounds with fine cotton instead of with charpie. 5th. His great experience in performing the operation.

Most of the calculi, which were exhibited to the Royal Society of Physicians of Vienna, were analyzed by Professor Kletzinsky, and were found to be composed of uric acid, oxalates, and triple phosphates.

IX.—*A Statistical View of Operations on the Tongue, and more Especially in Reference to their Danger from Hemorrhage.* By OTTO JUST, JR., M.D., of Zittau. (Schmidt's Jahrbücher, and London Medical Review, January, 1861.)

Dr. Just divides the different methods of removing the tongue into the following groups: A. *Operations with the knife.* 1. Amputation or partial excision. 2. Amputation by flap-incision, or wedge-like excision. 3. Total amputation or extirpation. B. *Ligature.* C. *Ecrasement lineaire.* D. *Cauterization.* 1. By the actual cautery, galvanocaustic. 2. By the potential cautery.

A. *Operations with the knife.* 1. *Amputation.*—Dr. Just has collected thirty-three cases of partial removal of the tongue for carcinoma, and sixteen for hypertrophy. To render the organ more accessible to the knife, as well as to ligature the vessels with more facility, it should be fixed by forceps, or by a stout ligature carried through its entire thickness. When the incisions have to extend toward the base of the tongue, on account of the small space we have to operate in, the procedure becomes more difficult and the arrest of hemorrhage is not so easy. In these cases more room can be gained by slitting open the cheek of the affected side, or by drawing out the tongue through an aperture in the neck, or by sawing through the symphysis of the lower jaw. These methods will, however, be unnecessary if the lingual arteries be previously ligated. 2. *Wedge-like excision.*—Under this head are mentioned twelve cases of cancer, one of telangiectasis, and eleven of hypertrophy. 3. *Extirpation.*—The author considers this operation justifiable when the whole of the tongue is diseased. Of three cases, one recovered.

*Methods of stopping the hemorrhage.*—The following means were employed for the arrest of hemorrhage, in sixty-two cases in which it is mentioned: In 29 cases, ligature of the vessels in the wound; in 2, ligature *en masse;* in 7, prophylactic ligature of the lingual artery; in 7, the actual cautery; in 6, ice and cold water; in 7, suture of the edges of the wound after flap-incision; in 3, styptics; in 1, bleeding spontaneously stopped. Ligature of the arteries in the wound is the safest method, and has never been followed, when employed alone, by secondary hemorrhage. The actual cautery, cold or styptics should be restricted to slight cases. Secondary hemorrhage ensued rather frequently when the actual cautery had been applied. Suture of the edges of the wound is unsafe, for in three-sevenths of the cases it was followed by secondary bleeding. Ligature of the lingual artery above the hyoid bone should only be resorted to when the base of the tongue is diseased and the mouth of large size; two of these cases died of pyæmia.

*Period of recovery.*—The most rapid recoveries took place when sutures had been used after excision of a wedge-shaped piece; in sixteen of these cases, fourteen united in from six to fourteen days. After amputation in general, it required from twelve days to four weeks. The cases treated by sawing through the symphysis took from one to two months.

*Result.*—Eight of sixty-eight cases terminated fatally, being 11¾ per cent. In five cases death resulted from pyæmia.

B. *Removal by ligature.*—Nineteen cases are quoted of ligature for cancer, and seven for hypertrophy; and two of these ended fatally, one frôm pyæmia, and one from poisoning by the offensive discharge. This method is troublesome and disgusting from the very fetid discharge, occasioning the patient great misery. Gangrene commences in about twenty-four hours after the application of the ligature, and on an average eleven days are required for the separation of the mass. The wound left after the detachment of the slough will heal generally in about fourteen days. Hemorrhage occurred in three instances, twice from too early a separation of the ligature, and once from the wound made for the passage of the thread.

C. *Amputation by means of écrasement lineaire.*—The écraseur has answered an excellent purpose in removal of the tongue, hemorrhage being effectually prevented when the instrument is made to act slowly. Twenty-one cases were treated by this method, and all recovered, the period of convalescence averaging three weeks.

D. *Amputation by means of the cautery.* 1. By the *galvanic cautery.*—Eight cases come under this head, hemorrhage attending four. The vessels required ligation in the wound twice, and once after previous ligation of the lingual artery. Secondary hemorrhage followed in about one-third of the cases. To prevent bleeding, the wire should be thick, the galvanic current weak, and the wire of the loops should not be heated beyond a black heat. 2. *Extirpation of the whole tongue by means of the potential cautery.*—One case only is given, in which the organ was destroyed by means of chloride of zinc.

The author has collected seventy-two cases of cancer of the tongue, in forty-nine of which the subjects were males, in twenty-three females; the frequency in males being pretty nearly in the ratio of five to two. In sixty-three cases the age is noted, showing that it occurs most frequently between forty and sixty years. In fifty-five cases the seat of the disease was, in seven, the tip of the tongue; in nine, the whole breadth of the organ; in nineteen, the right; and in twenty, the left side. In seventy-two cases there were eighteen recurrences; but the number is too small, as many of the cases were published soon after the operation.

To sum up the different methods of treatment, we find that the knife is especially adapted for removing small portions near the tip of the organ; the ligature has but one advantage, that of preventing hemorrhage, and the écraseur has shown more favorable results than those of the knife.

### X.—*Cases of Trephining in Syphilitic Disease of the Bones of the Skull.* By HENRY LEE, F.R.C.S. (Beale's Archives of Medicine, No. 6, 1860.)

In cases of syphilitic disease of the bones of the skull, the outer and middle tables are alone generally carious or necrosed, or the internal table may undergo the same changes, without the external being similarly affected. In the latter case, however, the internal table is affected to a small extent only, and usually in spots where the outer plate has already perished, and it occasionally leads to effusions on the dura mater, which are confined by the outer tables.

When the internal table is diseased, the only means of preventing the progress of the malady is the removal of the carious or necrosed bone; and if but a small portion be taken away, the irritation of the membranes of the brain will be relieved in a marked degree. A prominent symptom in the cases quoted by Mr. Lee, an abstract of two of which we give below, was the extensive, persistent, and recurring ulceration of portions of the skin, occurring upon the side upon which the cranial bones were principally diseased.

*Case* I.—Mary B., aged twenty-four, stated, on her admission into the Lock Hospital, that, six years previously, she had contracted syphilis, followed by secondary symptoms. Six months ago, she noticed a soft swelling on her forehead, which gave way and denuded the bone. She was very weak, much emaciated, and unable to walk without assistance. A portion of the frontal bone, about three inches in diameter, was exposed and necrosed. Several nodes existed on the head, one of which had burst over the right parietal bone, and another over the anterior extremity of the saggital suture. There was an extensive ulcer under the chin and on the left side of the cheek, which had completely detached the lobe of the ear. She was unable to hear, except when spoken to very loudly, had lost the sense of smell, and the sensibility of the right side of the face, including the right half of the upper lip, was greatly impaired. On the twenty-ninth of February a node over the right clavicle discharged itself, exposing the bone, and on the fifth of March she could walk about the wards; but the ulcer on the neck was enlarging. Three days subsequently, the trephine was applied near the centre of the exposed frontal bone, and its entire thickness removed. The dura mater was opaque, but bled at a few points; the internal table of the bone was soft and vascular, much roughened, and presented a worm-eaten appearance. The middle and external plates had lost their vitality. From March ninth to March fourteenth, on walking, she had strabismus of the right eye, which lasted two or three hours. During the next month she gained flesh rapidly, the sensibility of the cheek had returned, the hearing was restored, the ulcers gave no pain, and she was able to walk about the garden. On the first of May the ulcer of the neck was healed, and up to July a slight numbness of the right half of the upper lip still remained.

*Case* II.—A tradesman presented himself to Mr. Lee in March, 1859, with an induration of the upper part of the root of the penis. Eight years previously, an ulcer appeared over the left eye, and at the same time the skin over the right elbow began to ulcerate. This extended, so as finally to involve the whole arm. Three years afterward he had a severe convulsive fit, and in 1857 it became evident that the cranial bones were affected. He was now seized with violent spasmodic contractions of the right side of the face, and four months later a second attack followed, lasting six hours and a half, and attended with partial paralysis of the right side of the body and tremor of the limbs. Subsequent to this, several milder attacks occurred. When admitted to the Lock Hospital, September 13th, 1860, the ulcer on the outer side of the right forearm still existed, the skin was firmly cicatrized from the shoulder to the wrist, with no motion in the elbow and wrist joints. The hand was œdematous, and the frontal and parietal bones were denuded in several places, and their outer tables extensively carious or necrosed. The trephine was applied on the twenty-fifth of October, at several points over the right parietal bone, and, where most diseased, the whole thickness of the bone removed. At other places, the outer and middle tables were alone removed. The dura mater bled freely, and was not covered by deposits, and the internal face of the bone was slightly eroded. On the fifth of November he had two fits similar to those before the operation, and three weeks later, another slight attack. From this time on to the twenty-third of December, when he was discharged, he had no more convulsions, the wounds of the scalp looked healthy, the ulcer of the arm had nearly healed, having been open between eight and nine years, and he had gained much in flesh and strength.

